# Inhibition of DRP1-dependent mitochondrial fission by Mdivi-1 alleviates atherosclerosis through the modulation of M1 polarization

**DOI:** 10.1186/s12967-023-04270-9

**Published:** 2023-06-30

**Authors:** Ze-da-zhong Su, Chun-qiu Li, Hua-wei Wang, Min-ming Zheng, Qing-wei Chen

**Affiliations:** 1 Department of General Practice, The Second Affiliated Hospital of Chongqing Medical University, Chongqing, China; 2 Department of Ophthalmology, The Second Affiliated Hospital of Chongqing Medical University, Chongqing, China

**Keywords:** Atherosclerosis, Mdivi-1, DRP1, Mitochondrial fission, M1 polarization, NLRP3 inflammasome

## Abstract

**Background:**

Inflammation and immune dysfunction with classically activated macrophages(M1) infiltration are important mechanisms in the progression of atherosclerosis (AS). Dynamin-related protein 1 (DRP1)-dependent mitochondrial fission is a novel target for alleviating inflammatory diseases. This study aimed to investigate the effects of DRP1 inhibitor Mdivi-1 on AS.

**Methods:**

ApoE^−/−^ mice were fed with a high-fat diet supplemented with or without Mdivi-1. RAW264.7 cells were stimulated by ox-LDL, pretreated with or without MCC950, Mito-TEMPO, or Mdivi-1. The burden of plaques and foam cell formation were determined using ORO staining. The blood lipid profles and inflammatory cytokines in serum were detected by commercial kits and ELISA, respectively. The mRNA expression of macrophage polarization markers, activation of NLRP3 and the phosphorylation state of DRP1 were detected. Mitochondrial reactive oxygen species (mito-ROS), mitochondrial staining, ATP level and mitochondrial membrane potential were detected by mito-SOX, MitoTracker, ATP determination kit and JC-1 staining, respectively.

**Results:**

In vivo, Mdivi-1 reduced the plaque areas, M1 polarization, NLRP3 activation and DRP1 phosphorylation at Ser616. In vitro, oxidized low-density lipoprotein (ox-LDL) triggered M1 polarization, NLRP3 activation and abnormal accumulation of mito-ROS. MCC950 and Mito-TEMPO suppressed M1 polarization mediated foam cell formation. Mito-TEMPO significantly inhibited NLRP3 activation. In addition, Mdivi-1 reduced foam cells by inhibiting M1 polarization. The possible mechanisms responsible for the anti-atherosclerotic effects of Mdivi-1 on reducing M1 polarization were associated with suppressing mito-ROS/NLRP3 pathway by inhibiting DRP1 mediated mitochondrial fission. In vitro*,* similar results were observed by DRP1 knockdown.

**Conclusion:**

Inhibition of DRP1-dependent mitochondrial fission by Mdivi-1 alleviated atherogenesis via suppressing mito-ROS/NLRP3-mediated M1 polarization, indicating DRP1-dependent mitochondrial fission as a potential therapeutic target for AS.

**Supplementary Information:**

The online version contains supplementary material available at 10.1186/s12967-023-04270-9.

## Introduction

The accumulation of lipid deposits in the vascular wall and local inflammation are the main factors determining the progression of atherosclerosis (AS) [1]. Although various cells are involved in this process, macrophages were shown to be a fundamental contributor [2]. It was reported that the polarization state of macrophages plays a paramount role in the development and progression of AS [3]. At the broadest level, macrophages can be mainly polarized into the classically activated macrophages (M1) and alternatively activated macrophages (M2) [3, 4]. IL-6 and tumor necrosis factor α (TNF-α) are synthesized and released by M1 polarized macrophages, which can promote plaque growth, while M2 polarized macrophages can prevent AS by decreasing the plaque size and is strongly associated with anti-inflammatory effects by releasing anti-infammatory factors such as IL-10. Thus, macrophage polarization has been regarded as a novel target to prevent and alleviate AS [3–5]

Mitochondria are critical organelles for reactive oxygen species (ROS) generation and energy production [6]. Mitochondrial morphologic change is a constant process dependent on the fusion and fission of the outer and inner membranes [7]. A recent study highlighted the important role of mitochondrial dynamics (fusion and fission) in maintaining mitochondrial function [8], which is seemingly regarded as beneficial in distributing metabolites, proteins and DNA and relying on the mitochondrial network. However, excessive mitochondrial fission might lead to mitochondrial fragmentation, impair the electron transport chain and cause abnormal accumulation of mitochondrial ROS (mito-ROS), which may induce injury to mammalian cells [9, 10]. Mitochondrial fission is mainly modulated by dynamin-related protein 1(DRP1) [11]. Furthermore, excessive mitochondrial fission plays a significant role in inflammatory airway diseases [12] and liver ischemia–reperfusion injury [13]. In AS, DRP1 dependent mitochondrial fission contributes to diabetes-associated AS progression by influencing the functional status of endothelial cells [14]. However, whether the inhibition of DRP1-dependent mitochondrial fission can efficiently prevent AS by regulating M1 macrophage polarization is yet undiscovered.

Nod-like receptor family pyrin domain containing 3 (NLRP3) inflammasome is intracellular supramolecular complexes comprising a sensor molecule (NLRP3), the adaptor apoptosis associated specklike protein and the effector pro-caspase-1. Upon activation, NLRP3 recruits apoptotic speck like protein resulting in formation of the so called ASC speck, which acts as a molecular platform for the activation of pro-caspase-1, and released cleaved-caspase-1. NLRP3 inflammasome has been recently shown to have a vital role in AS [15]. It was reported that the activation of NLRP3 could promote macrophages polarizing towards M1-like phenotypes [16, 17]. Mitochondrial division inhibitor-1 (Mdivi-1) is a small molecule inhibitor of mitochondrial fission by specifically targeting DRP1 to improve mitochondria function [18, 19], thus to have a protective effect on target cells. Inhibition of DRP1 by Mdivi-1 reduced proprotein convertase subtilisin/kexin type 9 (PCSK9) secretion in HepG2 cells with concomitant changes in macrophage polarization [20]. Moreover, DRP1 activation-induced mitochondrial fission aggravates myocardial injuries by activating NLRP3 inflammasome [21]. Maintaining mitochondrial dynamics can exert an anti-atherosclerotic effect by reducing NLRP3 related pyroptosis in endothelial cells [22]. Yet the role of Mdivi-1 on M1 polarization in AS remains unclear. Based on these, we hypothesized that DRP1 inhibitor Mdivi-1 might play a protective role against AS by inhibiting NLRP3 inflammasome mediated M1 polarization.

## Methods

### Mice

SPF healthy 8-weeks-old male C57BL/6 J ApoE^−/−^ mice (19–22 g) were provided by Chongqing Medical University Animal Center. The mice were kept at a temperature of 19–25 °C and relative humidity of 40–50%. 7 mice in each cage, they were given free water. Mdivi-1 was dissolved in DMSO and diluted with saline. After adaptive feeding for a week, ApoE^−/−^ mice were randomly separated into three groups. The mice (n = 7) in the high-fat diet (HFD) group were fed a western diet (1.25% cholesterol and 20% fat; 40% kcal as fat, Research Diebts, Beijing) + vehicle (an equal volume of DMSO + saline) for 12 weeks to induce AS. The mice (n = 7) in the control group were fed with normal chow + vehicle for 12 weeks. The mice in Mdivi-1 (MCE, USA) + HFD group were fed with the western diet and orally gavaged with Mdivi-1 (5, 10, 20 mg/kg/day, n = 7 for each dose respectively, sigma, USA) for 12 weeks [23–25]. The animal experimental protocol was reviewed and approved by Institutional Animal Care and Use Committee of the second affiliated hospital of Chongqing Medical university, Chongqing, China.

### Sample collection

After 12 weeks, 3% pentobarbital sodium was used to anesthetize the mice. To harvest the heart and aortic tissue (from the aortic root to common iliac artery branches), the mice were sacrificed by cervical dislocation and perfused with 1 × PBS through the left ventricle. Oil Red O (ORO) staining was performed with 4% paraformaldehyde to fix the arteries of mice from each group for 24 h. The remaining carotid arteries were used for western blot and quantitative real-time PCR.

### Cell culture

RAW264.7 cells were obtained from the American Type Culture Collection (ATCC, USA) and were grown in Dulbecco’s modified Eagle medium (DMEM, Gbico, USA), supplemented with 10% FBS (Gbico, USA) and 1% penicillin at 37 °C with 5% CO_2_ and 95% air. Macrophages were seeded in 6-well, 12-well or 96-well plates. For experiments involving pharmacological inhibitors (inhibitors dissolved in DMSO were used to treat cells, and an equal volume of DMSO was used as the vehicle.), the macrophages were preprocessed with 10 μM MCC950 (MCE, USA), 100 μM Mito-TEMPO (Sigma, USA) or Mdivi-1 (25, 50, 75 μM, MCE, USA) [20, 26, 27] for 2 h and subsequently treated with oxidized low-density lipoprotein (ox-LDL) (Yiyuan Biotechnologies, China) at a concentration of 50 μg/ml for 24 h in the presence of these inhibitors. In control group, RAW264.7 cells were treated with vehicle. In ox-LDL group, RAW264.7 cells were treated with 50 μg/ml ox-LDL + vehicle. After different treatments, the RAW264.7 cells were harvested and subjected to subsequent experiments.

### ORO staining

After interventions, 1 × PBS was used to wash the cultured RAW264.7 cells 3 times and fixed with 4% paraformaldehyde for 10 min. Then, the cells were washed twice and subsequently stained with ORO staining reagents for 30 min. After staining, the cells were thoroughly washed with 1 × PBS until no redundant ORO working solution remained. Lipid droplets in the cells were observed and photographed using an inverted microscope.

To detect the lipid deposition within the arterial wall and aortic sinus plaque, the fixed heart and aortic tissues were kept at − 80 °C and soaked in an optimum cutting temperature compound. Then, serial cryosections were obtained, and ORO staining was performed following the manufacturer’s instructions (Solarbio, China).

### Measurement of blood lipid profles

Blood samples were centrifuged (3000 rpm for 10 min) to harvest serum, which is stored at − 80 °C. The blood lipid profles including total cholesterol (TC), low density lipoprotein cholesterol (LDL-C) and high density lipoprotein cholesterol (HDL-C) were measured by commercial kits according to the manufacturer’s instructions (Ruixin biotech, China).

### Detection of serum cytokines and oxidative stress biomarkers

Inflammatory cytokines (TNF-α, IL-6 and IL-10) were detected by enzyme-linked immunosorbent assay (ELISA, Jianglai, China) according to manufacturer’s protocols. The SOD activity in macrophages and level of MDA in cell supernatant were measured by commercial kits following the manufacturer’s instructions (Beyotime, China).

### Immunohistochemical Staining

After soaking with antigen retrieval solution, the aortic sections were dried at 60 °C for 30 min. 3% H2O2 was used to to block the endogenous peroxidase activity and then goat serum was used to incubate the sections for 15 min at room temperature. Subsequently, primary antibodies (rabbit anti-CD86, Affinity, China, #DF6332; rabbit anti-CD206, CST, USA, #24595) were added at 4 °C overnight. After that, the sections were incubated with Goat anti-Rabbit IgG (1:500) for 2 h. The sections were captured with an inverted microscope. The mean intensity of staining area was analyzed by Image J.

### Quantitative Real-Time PCR (qRT-PCR)

The TRIzol reagent (TaKaRa, Japan) was used to extract total RNA from the aortic tissue and macrophages. A PrimeScript RT reagent kit (Accurate Biotechnology, China) was used to harvest cDNA by reverse transcription, and the mRNA expression levels were determined. SYBR Green Premix Pro Taq qPCR kit(Accurate Biotechnology, China) was used to perform PCR with iNOS, CD86, IL-6, TNF-α, MCP-1, CD206, CD163, IL-10 and β-actin specific primers using a CFX96 Real-Time PCR Detection System (Bio-Rad, USA).

The specific primer sequences are listed in Additional file 1: Table S1. The 2^−△△^Ct method was used to detect differences in gene expression among the different groups. qRT-PCR was conducted for three independent experiments, with β-actin as the reference gene.

### Mitochondrial staining of macrophages

MitoTracker Green reagent (Beyotime, China) was used to determine the number of mitochondria in the macrophages. After various interventions, the macrophages were washed 3 times with 1 × PBS and incubated with the MitoTracker Green reagent for 30 min. Finally, after 3 times with 1 × PBS, Hoechst staining was performed to stain the cells for 10 min and imaged under a Nikon A1 confocal microscopy. The number of mitochondria was counted by Image J and the average number of mitochondria per cell was analysed [22].

### ATP detection

An ATP assay kit (Beyotime, China) was used to quantify the ATP level following the manufacturer’s instructions. The Varioskan LUX Multimode Reader (Thermo Fisher, USA) was applied to record luminescence.

### Mitochondrial membrane potential (∆Ψm)

The macrophages were stained with JC-1 following the manufacturer’s instructions of the JC-1 staining assay kit (Servicebio, China). In brief, after interventions, cultured RAW264.7 cells were washed by 1 × PBS 3 times and stained with JC-1 staining solution for 20 min at 37 ℃. The absorption of monomer (fluorescence excitation set at 490 nm; fluorescence excitation set at 530 nm) and J-aggregates (fluorescence excitation 525 nm; fluorescence excitation 590 nm) was detected by a modular multi-technology microplate reader (Thermo Scientific™ Varioskan™ LUX, USA) with fluorescence spectrophotometry following the manufacturer’s protocol. The mitochondrial membrane potential difference between groups was reflected by the JC-1 ratio (aggregates/monomer).

### Subcellular fractionation

Macrophages were collected after different treatment. MinuteTM Cytoplasmic Extraction Kit (Invent Biotechnologies, USA) and MinuteTM Mitochondria Isolation Kit (Invent Biotechnologies, USA) were used to isolate cytosol fractions and the mitochondria fractions, respectively.

### siRNA transfection

According to instruction, RAW264.7 cells were transfected with DRP1 siRNA, or control siRNA by employing GP-transfect-Mate reagent (Shanghai GenePharma Co.,Ltd). After siRNA transfection, the cells were treated with ox-LDL for 24 h.The specific sequences are listed as follows:

Negative control:

Sense: UUCUCCGAACGUGUCACGUTT; Antisense: ACGUGACACGUUCGGAGAATT;

DRP1 siRNA:

Sense: GUGUCCCAAAGGCAGUAAUTT; Antisense: AUUACUGCCUUUGGGACACTT.

### Detection of mito-ROS

The intracellular mito-ROS level was detected by mito-SOX Red Mitochondrial Superoxide Indicator (ABclonal, China). After incubation with diluted mito-SOX for 10 min at 37 ℃, macrophages were washed by 1 × PBS 3 times and the fluorescence intensity was detected with an inverted microscope.

### Western Blot

The tissues or cells were lysed in RIPA (Bestbio, China) with a protease inhibitor cocktail (Beyotime, China). Protein concentrations were detected with a BCA protein assay (Beyotime, China). Equal amounts of denatured proteins (30 μg) were run on 12% SDS-PAGE, which were then transferred to polyvinylidene difluoride (PVDF) membranes (Millipore, USA). The membranes were blocked at room temperature by 5% nonfat milk for 1 h. The blots were subsequently incubated with rabbit anti-DRP1 (1:1000, CST, USA, #8570 s), rabbit anti-phosphorylation of Drp1(Ser616) (1:1000, Absin, China, abs137991), rabbit anti-NLRP3 (1:1000, CST, USA, #15101 s), rabbit anti-caspase-1 (1:1000, Affinity, China, #AF5418), rabbit anti-Cleaved-Caspase 1 (1:1000, Affinity, China, #AF4005), rabbit anti-VDAC1 (1:2000, CST, USA, #4866 s) and rabbit anti-β-actin (1:5000, CST, USA, #4970 s) primary antibodies at 4 °C overnight. Then, a fluorescent secondary antibody (1:10000, Jackson, USA, #111–035-003) was then used to incubate the membranes for 2 h at 4 °C. An ECL Kit (Beyotime, China) was used to detect protein bands, which were visualized using a Bio-Rad imaging system (Bio-Rad, USA).

### Statistical analysis

All data are expressed as the mean ± SD. Statistical analyses were performed using the GraphPad Prism 8.0 software (GraphPad, San Diego, CA, United State). The Student’s unpaired t-test was used to determine differences between two groups, and the one-way analysis of variance (ANOVA) test was used to compare multiple groups with post hoc Tukey’s test. *P* < 0.05 was considered statistically significant.

## Results

### Mdivi-1 alleviated AS lesions in HFD ApoE^−/−^ mice.

**Fig. 1:**
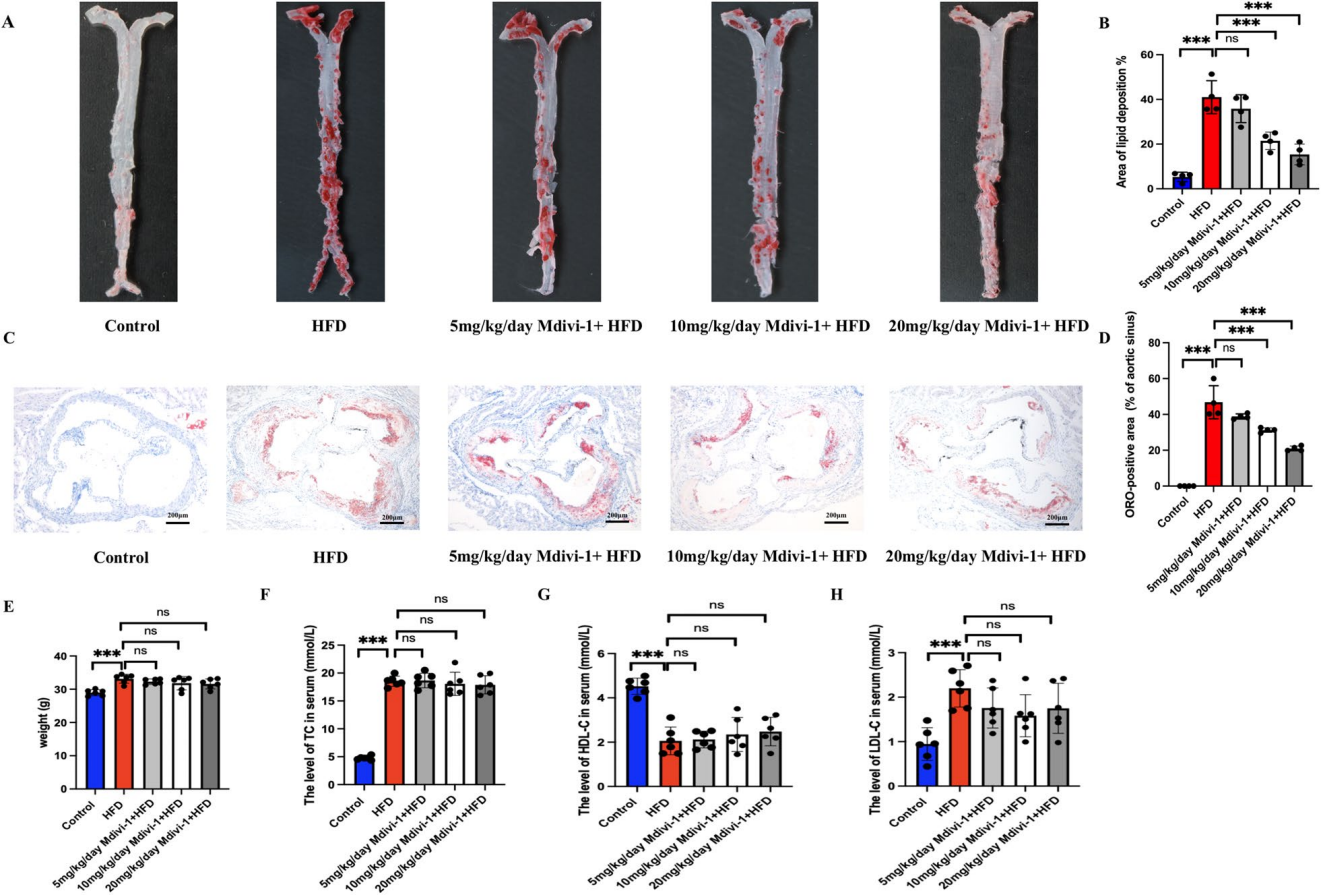
Mdivi-1 protected against atherosclerosis in HFD ApoE^−/−^ mice. The size of the aortic lesion (**A**, **B**) and the lipid deposition in the aortic sinus (**C**, **D**) determined by ORO staining (scale bar: 200 μm. 40 × magnification), n = 4. (**E**) Body weight determined by electronic scale, n = 6. The levels of TC (**F**), LDL-C (**G**) and HDL-C (**H**) in serum detected by the corresponding kits, n = 6. * *p* < 0.05, ** *p* < 0.01, *** *p* < 0.001, and ns *p* > 0.05

To determine the role of Mdivi-1 (5, 10, 20 mg/kg/day) in AS, the plaque area in HFD ApoE^−/−^ mice was measured. The results showed that the extent of AS lesions was significantly reduced in the Mdivi-1(20 mg/kg/day) + HFD group compared with the HFD group (Fig. 1A, B). The ORO staining data suggested that Mdivi-1(20 mg/kg/day) could significantly decrease lipid deposition in the aortic sinus (Fig. 1C, D). Body weight increased in HFD group compared with the control group but did not change significantly after the Mdivi-1 (5, 10, 20 mg/kg/day) treatment (Fig. 1E) and similar results were observed in the levels of blood lipid profles including TC, HDL-C and LDL-C (Fig. 1F, G, H).

### Mdivi-1 effectively alleviated AS lesions by inhibiting M1 polarization

**Fig. 2:**
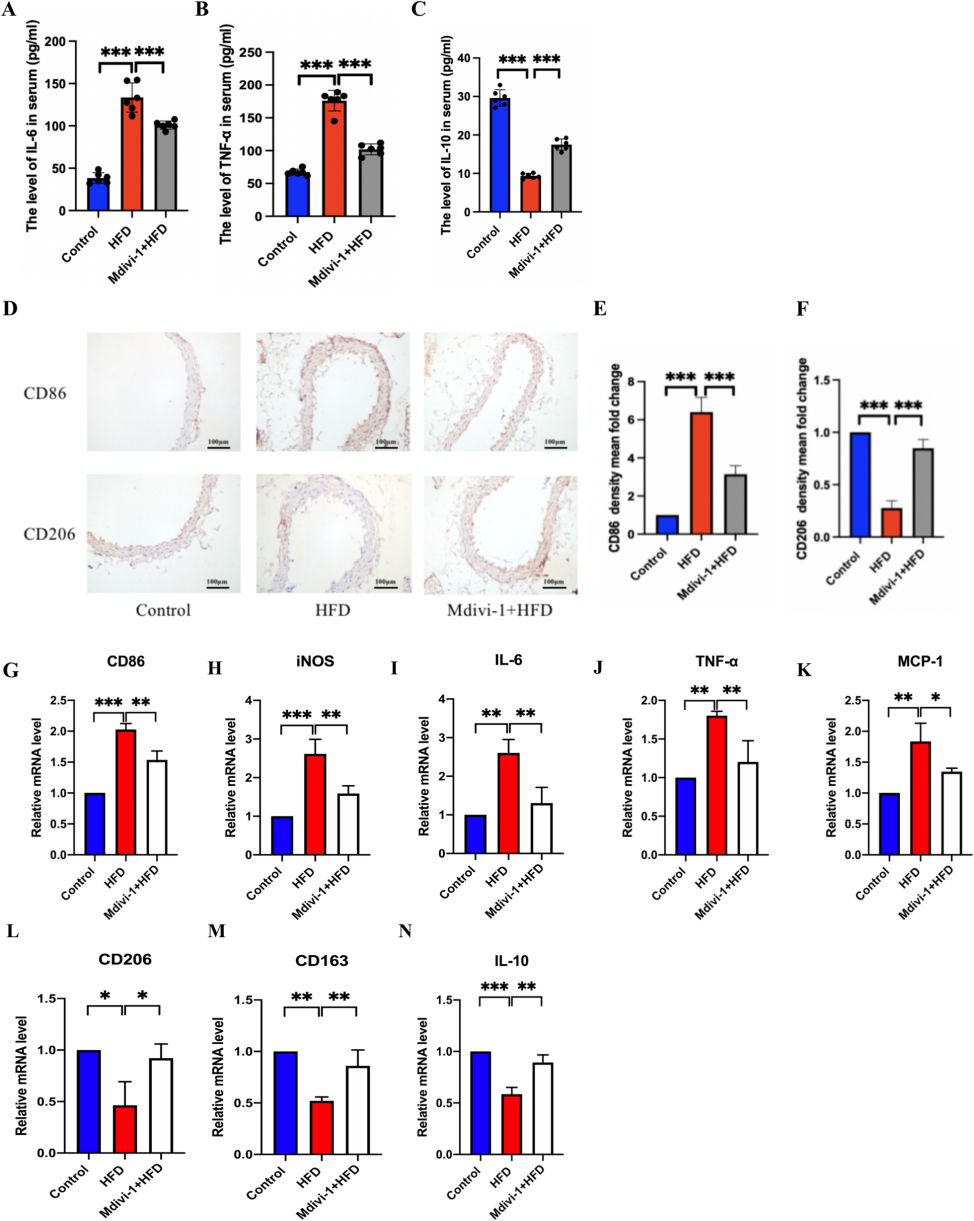
Mdivi-1 regulated M1 polarization in aortic tissue from HFD ApoE^−/−^ mice. The levels of inflammatory cytokines including IL-6 (**A**), TNF-α (**B**), and IL-10 (**C**) in the serum measured by ELISA, n = 6. (**D**) Representative immunohistochemical staining showing the expression of CD86 and CD206 in aortic sections (scale bar: 100 μm. 200 × magnification). (**E,F**) The mean density of CD86 and CD206 was analyzed, n = 3. The M1 markers mRNA, including CD86 (**G**), iNOS (**H**), IL-6 (**I**), TNF-α (**J**), MCP-1 (**K**) and M2 markers mRNA, such as CD206 (**L**), CD163 (**M**), IL-10 (**N**) detected by qRT-PCR, n = 3. * *p* < 0.05, ** *p* < 0.01, and *** *p* < 0.001

Considering that the progression of AS lesions is strongly associated with M1 polarization [3], the states of macrophage polarization in aortic tissues were detected to confirm whether Mdivi-1 inhibited plaque formation by modulating M1 polarization. The results showed that the levels of pro-inflammatory cytokines (IL-6, TNF-α) in serum from HFD mice were higher compared with control group and Mdivi-1 (20 mg/kg/day) treatment down-regulated IL-6 and TNF-α levels (Fig. 2A, B). On the contrary, the levels of IL-10 were decreased compared with control group and Mdivi-1 (20 mg/kg/day) treatment up-regulated IL-10 levels (Fig. 2C). The content of M1 marker CD86, in aortic tissue of model group increased significantly compared with control group, and it exhibited an obvious decrease after Mdivi-1 treatment (Fig. 2D, E). In addition, the content of M2 marker CD206, in aortic tissue of model group decreased significantly compared with control group, and it exhibited an obvious increase after Mdivi-1 treatment (Fig. 2D, F). The mRNA expression of M1 polarization-associated markers (CD86 and iNOS) was significantly higher in the HFD group aortic tissue compared with the control group and exhibited an obvious decrease after Mdivi-1 (20 mg/kg/day) treatment (Fig. 2G, H). As shown in Fig. 2 I, J, K, the mRNA levels of IL-6, TNF-α and MCP-1 were increased in the HFD group compared with the control group, but they were significantly reduced after Mdivi-1 (20 mg/kg/day) intervention. In addition, our data indicated that the mRNA expression of M2 markers (CD206, CD163 and IL-10) was reduced in the HFD group compared with the control group and they were increased after Mdivi-1 (20 mg/kg/day) intervention (Fig. 2L, M, N). These results demonstrated that Mdivi-1 could alleviate AS by inhibiting M1 polarization.

### Inhibition of phospho-DRP1(Ser616) by Mdivi-1 protected against AS plaques by inhibiting NLRP3 activation in aortic tissues of HFD mice

**Fig. 3:**
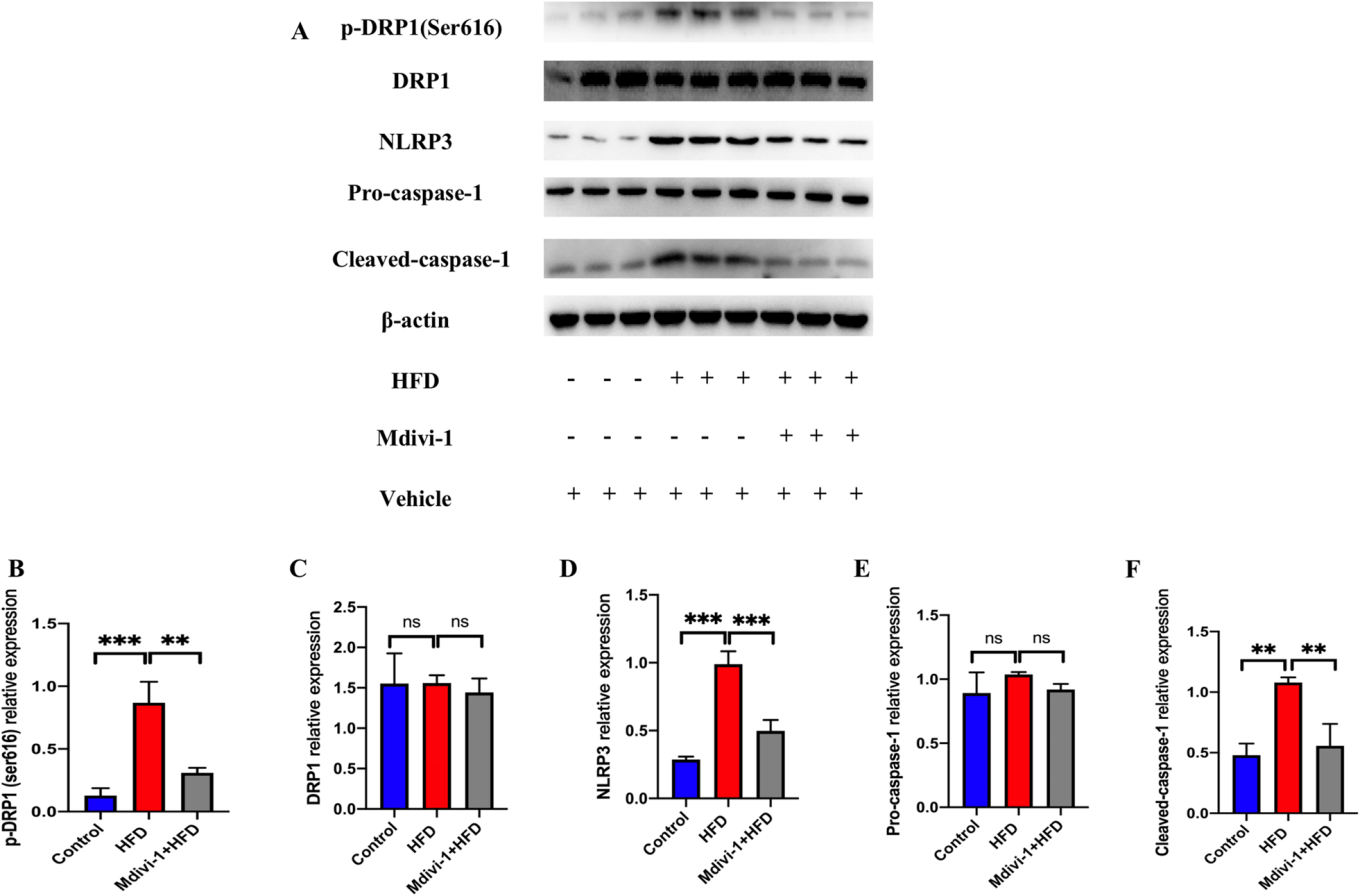
Mdivi-1 reduced phosphorylation of DRP1 (Ser616) and NLRP3 activation in the aortic tissues of HFD ApoE^−/−^ mice. Western blot showing the protein level of phosphorylation-DRP1 (Ser616), total DRP-1, NLRP3, pro-caspase-1, and cleaved-caspase-1 (**A**). The relative protein expression levels of (**B**) phosphorylation-DRP1 (Ser616)/β-actin, (**C**) DRP1/β-actin, (**D**) NLRP3/β-actin (**E**) pro-caspase-1/β-actin and (**F**) cleaved-caspase-1/β-actin in aortic tissues. Three independent replications were performed. ** *p* < 0.01, *** p < 0.001, and ns *p* > 0.05

NLRP3 activation is closely with AS progression [28]. Mdivi-1, an inhibitor of DRP1, was recently found to alleviate inflammation-related diseases by inhibiting NLRP3 inflammasome [29, 30]. However, the protective effects of Mdivi-1 in AS are poorly understood. To investigate the effects and probable underlying mechanism, we detected the protein levels of phospho-DRP1 (Ser616), DRP1, NLRP3, pro-caspase-1, and cleaved-caspase-1. In the HFD group, the levels of phospho-DRP1 (Ser616), NLRP3, and cleaved-caspase-1 were found to be higher than in the control group but were significantly downregulated after Mdivi-1 (20 mg/kg/day) treatment (Fig. 3A–F). The role of Mdivi-1 on mitochondrial fission in aortic tissue in HFD mice was shown in Additional file 2: Fig. S1.  These results indicated that Mdivi-1 could alleviate AS by inhibiting the NLRP3 inflammasome. However, the specific mechanism should still be explored in vitro.

### Ox-LDL triggers M1 polarization, NLRP3 activation and abnormal accumulation of mito-ROS in RAW264.7 cells

**Fig. 4:**
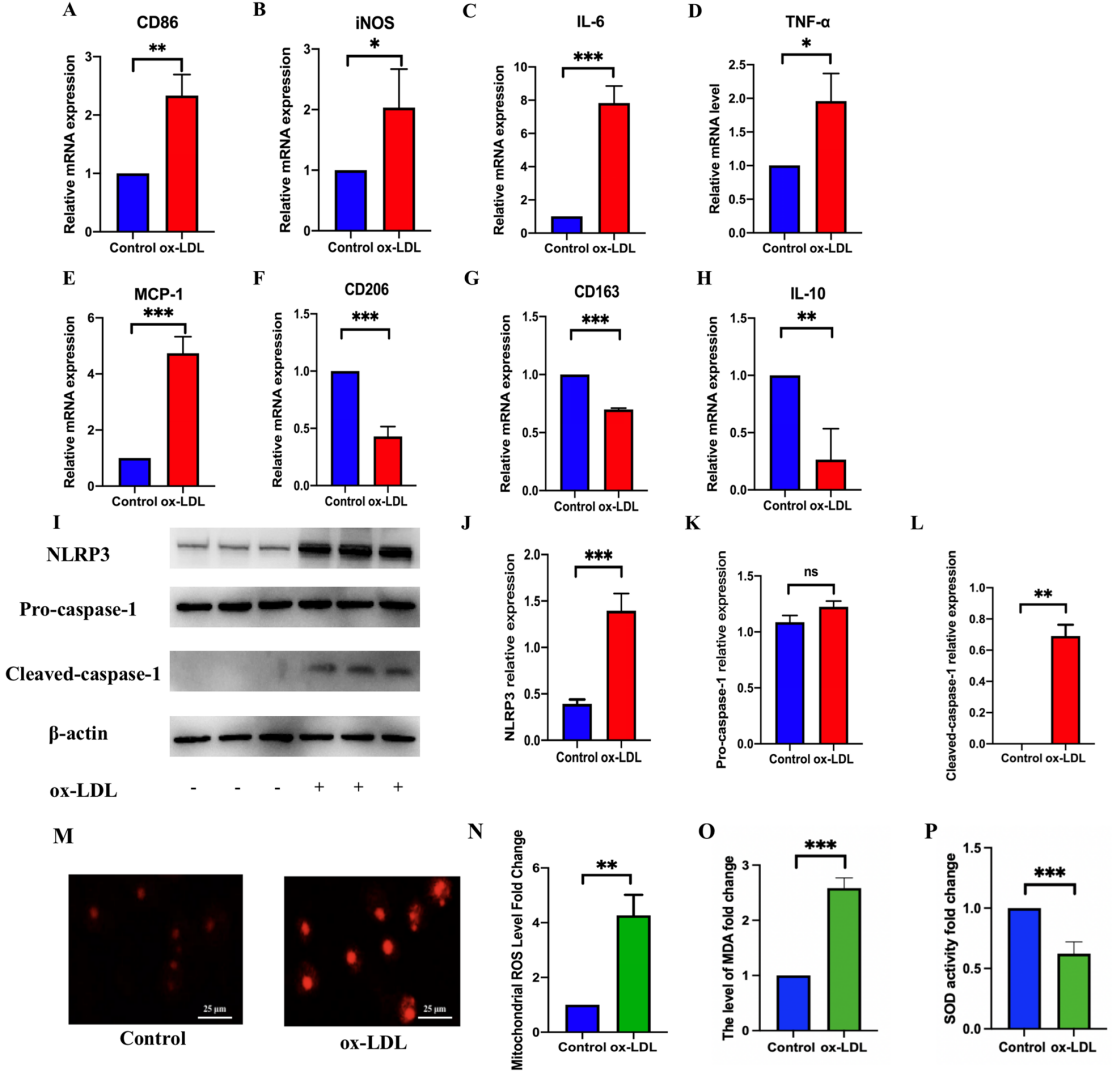
ox-LDL induced M1 polarization, NLRP3 activation, and abnormal accumulation of mito-ROS. qRT-PCR showing M1 polarization markers mRNA expression, including CD86 (**A**) and iNOS (**B**), and pro-inflammatory factors mRNAs such as IL-6 (**C**), TNF-α (**D**), and MCP-1 (**E**). RT-PCR indicating M2 markers mRNA expression including CD206(**F**), CD163(**G**) and IL-10(**H**). Western blot demonstrating the protein expression of NLRP3, pro-caspase-1, and cleaved-caspase-1 (**I**). The relative protein expression levels of (**J**) NLRP3/β-actin, (**K**) pro-caspase-1/β-actin and (**L**) cleaved-caspase-1/β-actin in RAW264.7 cells. Using mito-SOX to detect mito-ROS (**M,N**) (scale bar: 25 μm. 200 × magnification). The SOD activity and level of MDA detected by corresponding kits (**O,P**). Three independent replications were performed. * *p* < 0.05, ** *p* < 0.01, *** p < 0.001, and ns *p* > 0.05

To confirm the effects of ox-LDL on macrophage polarization and the underlying mechanism, 50 μg/ml ox-LDL was used to stimulate RAW264.7 cells. In response to ox-LDL, the mRNA expression of M1 markers (CD86 and iNOS) was significantly higher than the control group (Fig. 4A, B), and the mRNA level of pro-inflammatory cytokines (IL-6, TNF-α and MCP-1) was increased (Fig. 4C–E) compared with the control group. In addition, the expression of M2 markers in mRNA level (CD206, CD163 and IL-10) was inhibited in RAW264.7 cells induced by ox-LDL (Fig. 4F, G, H). Further, the ox-LDL treatment increased the protein expression of NLRP3 and cleaved-caspase-1 compared with the control group (Fig. 4I–L) and led to an lower activity of SOD (Fig. 4O) and an increased level of mito-ROS and MDA (Fig. 4M, N, P) compared with the control group.

### Mito-ROS/NLRP3 pathway mediated M1 polarization and associated foam cell formation

**Fig. 5:**
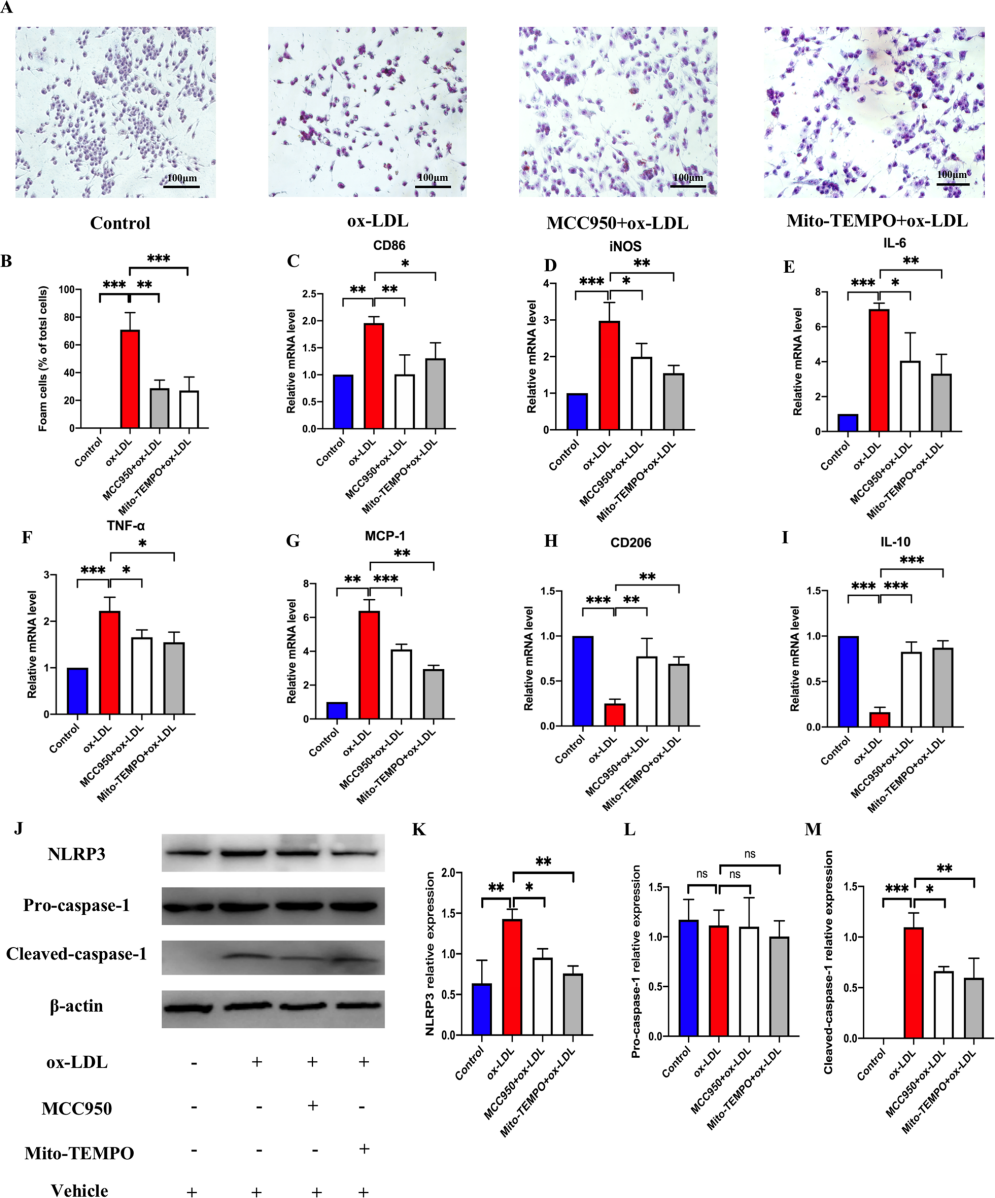
Mito-ROS/NLRP3 pathway mediated M1 polarization and foam cell formation. **A**, **B** ORO staining detecting lipid deposition in macrophages (scale bar: 100 μm. 200 × magnification). The mRNA expression of M1 markers, including CD86 (**C**) and iNOS (**D**), IL-6 (**E**), TNF-α (**F**), MCP-1 (**G**) and M2 markers, including CD206 (**H**) and IL-10 (**I**) detected by qRT-PCR. The protein level of NLRP3, pro-caspase-1, and cleaved-caspase-1 detected by western blot (**J**). The relative protein expression levels of (**K**) NLRP3/β-actin, (**L**) pro-caspase-1/β-actin and (**M**) cleaved-caspase-1/β-actin in RAW264.7 cells. Three independent replications were performed. * *p* < 0.05, ** *p* < 0.01, *** p < 0.001, and ns *p* > 0.05

NLRP3 inflammasome activation can trigger M1 polarization and induce several inflammatory diseases [17, 31, 32]. Further, it was reported that NLRP3 could be activated by abnormal ROS accumulation [33, 34], which is mainly produced by dysfunctional mitochondria [35]. However, whether the mito-ROS/NLRP3 pathway-mediated M1 polarization can promote AS is yet to be determined. Our results showed that both mito-ROS scavenger, Mito-TEMPO, and NLRP3 inhibitor, MCC950, reduced foam cell formation (Fig. 5A, B). The mRNA expression of M1 markers (CD86 and iNOS) (Fig. 5C, D) and pro-inflammatory factors (IL-6, TNF-α, and MCP-1) (Fig. 5E, F, G) expression were significantly downregulated by the two inhibitors. In addition, the mRNA expression of M2 markers (CD206 and IL-10) (Fig. 5H, I) expression were upregulated by the two inhibitors. Then, we further explored the specific molecular mechanisms via which MCC950 and Mito-TEMPO inhibited foam cell formation. As shown in (Fig. 5J–M), Mito-TEMPO and MCC950 inhibited the NLRP3 inflammasome activation by downregulating the protein levels of NLRP3, and cleaved-caspase-1 in RAW264.7 cells treated by ox-LDL. These results showed that the mito-ROS/NLRP3 pathway mediated M1 polarization promoted foam cell formation, and triggered AS.

### Mdivi-1 reduces M1 polarization-mediated foam cell formation by suppressing the mito-ROS/NLRP3 pathway

**Fig. 6:**
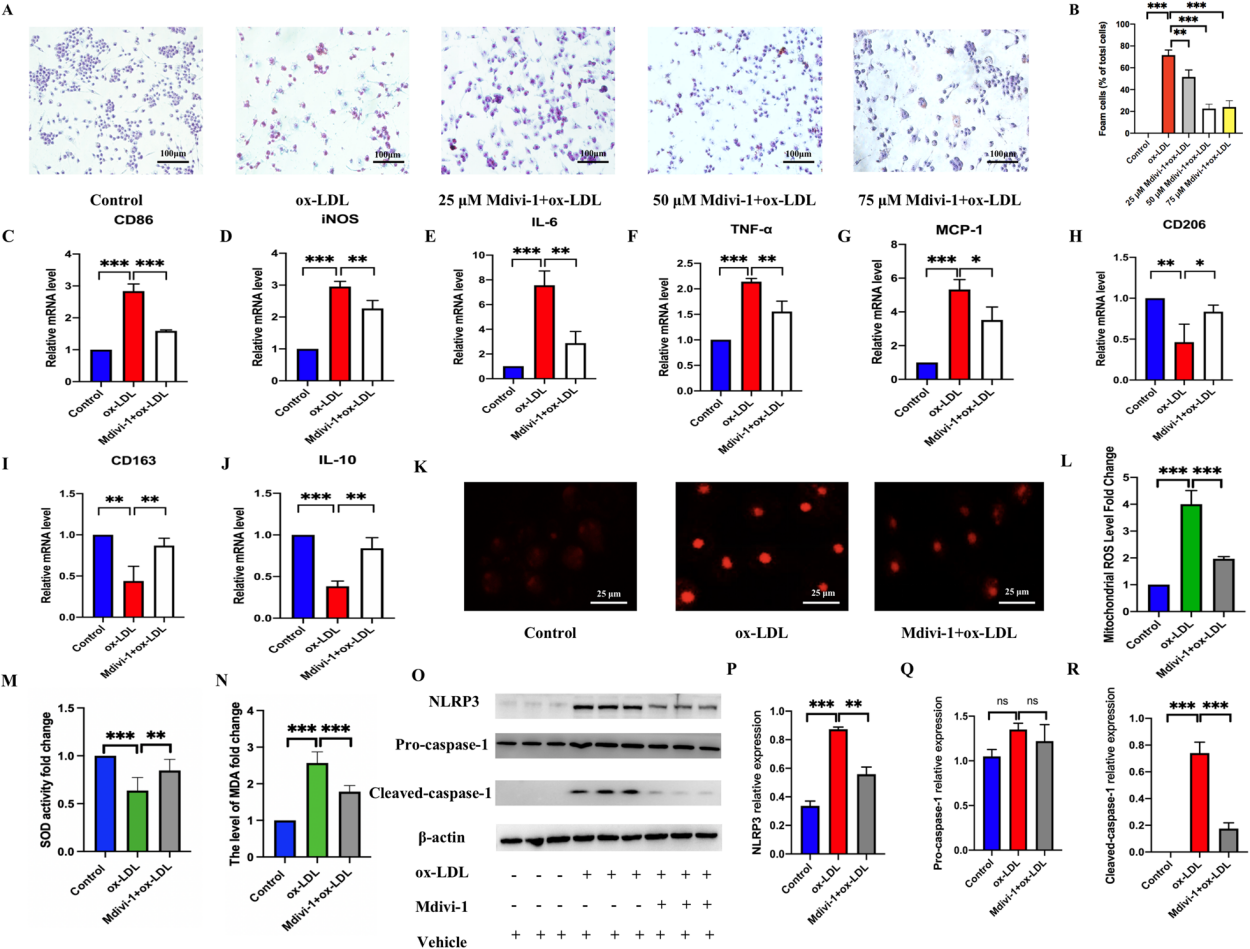
Mdivi-1 reduced M1 polarization mediated foam cell formation by inhibiting mito-ROS/NLRP3 activation. **A**, **B** ORO staining to detect lipid deposition in macrophages (200 × magnification). The mRNA expression of M1 polarization markers, including CD86 (**C**) and iNOS (**D**), IL-6 (**E**), TNF-α (**F**), MCP-1 (**G**) and M2 polarization markers, including CD206 (**H**), CD163(**I**) and IL-10 (**J**) detected by qRT-PCR. **K**, **L** Mito-ROS was detected by mito-SOX (scale bar: 25 μm. 200 × magnification). **M**, **N** The SOD activity and level of MDA detected by corresponding kits. The protein level of NLRP3, pro-caspase-1, and cleaved-caspase-1 detected by western blot (**O**). The relative protein expression levels of (**P**) NLRP3/β-actin (**Q**) pro-caspase-1/β-actin and (**R**) cleaved-caspase-1/β-actin in RAW264.7 cells. Three independent replications were performed. * *p* < 0.05, ** *p* < 0.01, *** p < 0.001, and ns *p* > 0.05

Here, we investigated the effects of inhibiting DRP1-dependent fission by Mdivi-1 (25, 50, 75 μM) on M1 polarization and its probable underlying mechanism. ORO Red staining (Fig. 6A) and percent positive foam cells (Fig. 6B) revealed that inhibition of DRP1-dependent fission by 50 μM Mdivi-1 significantly attenuated foam cell formation by reducing lipid droplets deposition. Then, 50 μM Mdivi-1 was used to improve foam cell formation by inhibiting M1 polarization, as evidenced by Mdivi-1-induced decrease of M1 markers (CD86, iNOS, IL-6, TNF-α and MCP-1) mRNA expression in RAW264.7 cells (Fig. 6C–G). M2 markers (CD206, CD163 and IL-10) expression increased in Mdivi-1 treated ox-LDL group (Fig. 6H, I, J). 50 μM Mdivi-1 improved the activity of SOD (Fig. 6M) and reduced MDA (Fig. 6N) and mito-ROS accumulation (Fig. 6K, L), which decreased the protein expression of NLRP3 and cleaved-caspase-1 (Fig. 6O, P, R) but pro-caspase-1 expression did not significantly change(Fig. 6Q). These results suggested that Mdivi-1 alleviated M1 polarization mediated foam cell formation by suppressing the mito-ROS/NLRP3 pathway.

### Mdivi-1 inhibited DRP1-dependent mitochondrial fission and improved mitochondrial dysfunction induced by ox-LDL

**Fig. 7:**
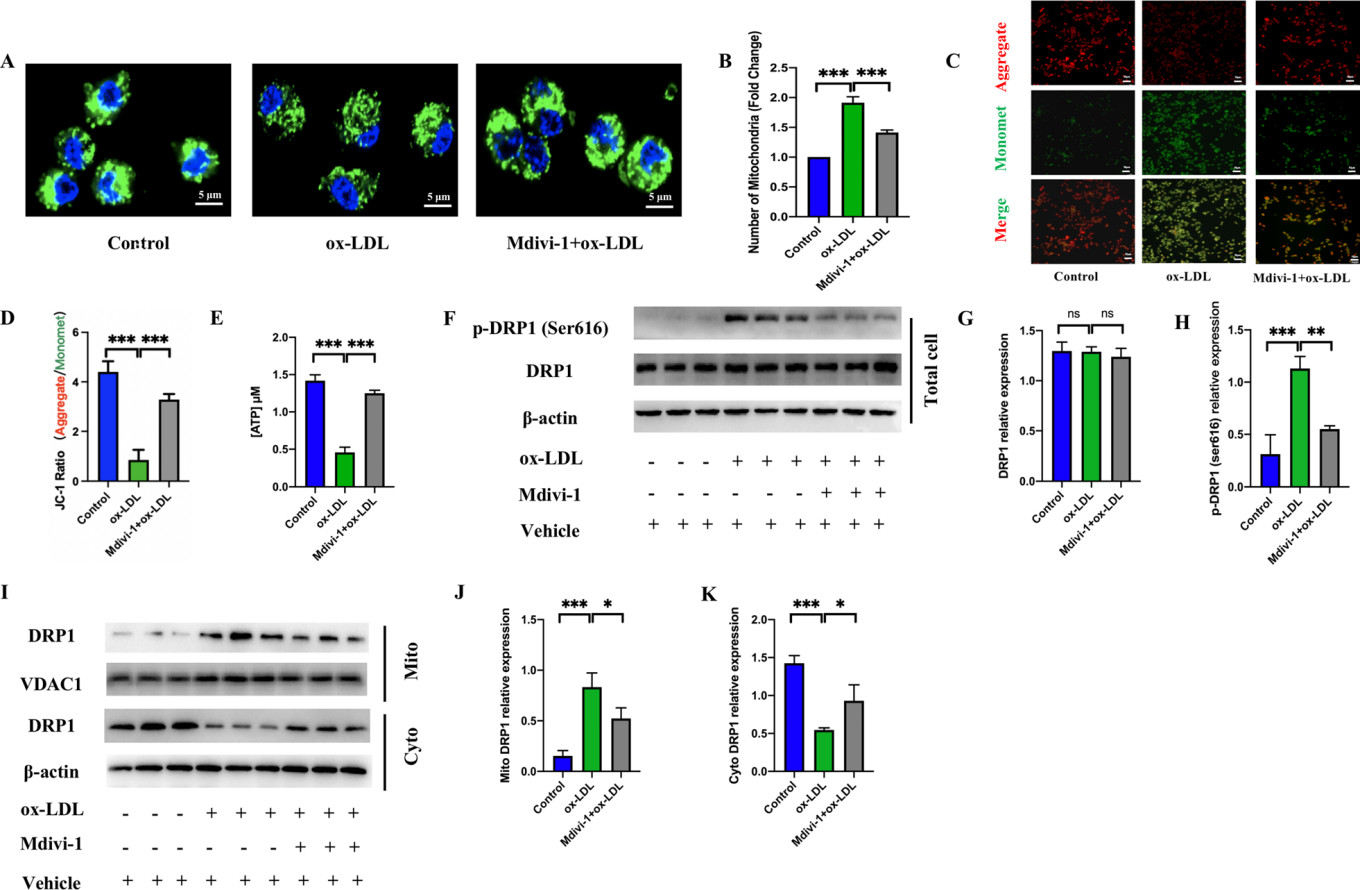
Mitochondrial fission inhibition by Mdivi-1 improved mitochondrial dysfunction caused by ox-LDL. **A** MitoTracker staining to detect the number of mitochondrial fragments (scale bar: 5 μm. 600 × magnification). **B** Analysis of the relative number of mitochondria. **C**, **D** JC-1 staining to detect mitochondrial membrane potential (scale bar: 50 μm. 200 × magnification). **E** Detecting ATP level by an ATP determination kit. **F** The protein level of phosphorylation-DRP1 (Ser616) and total DRP-1 detected by western blot. The relative protein expression levels of (**G**) DRP1/β-actin, and (**H**) phosphorylation-DRP1 (Ser616)/β-actin in RAW264.7 cells. (**I**) Western blot to detect the translocation of DRP1. The relative protein expression levels of (**J**) DRP1/VDAC1 in the mitochondria fractions and (**K**) DRP1/β-actin in cytosol fractions. Three independent replications were performed. * *p* < 0.05, ** *p* < 0.01, *** p < 0.001, and ns *p* > 0.05

Excessive mitochondrial fission lead to mitochondrial fragmentation, impair the electron transport chain and cause abnormal accumulation of mito-ROS [9, 10, 36]. DRP1 is an indispensable core molecule controlling mitochondrial fission and phosphorylation of DRP1 at serine 616 promotes DRP1 activity, which can stimulate DRP1 mitochondrial translocation [37] and promote fission. Recent studies have reported that Mdivi-1 could reduce the division of mitochondria by inhibiting the phosphorylation of DRP1 at Ser616 [18, 19, 38, 39]. Therefore, we explored the relationship between Mdivi-1 and mitochondrial function in macrophages induced by ox-LDL. Our results showed that the division of mitochondria was increased in the ox-LDL group, and 50 μM Mdivi-1 treatment could reduce the number of mitochondria by inhibiting fission (Fig. 7A, B). Unmerged figures are shown in Additional file 3: Fig. S2. JC-1 ratio and the ATP level were decreased after ox-LDL treatment, whereas, 50 μM Mdivi-1 improved the function of mitochondria by ameliorating mitochondrial membrane potential and ATP synthesis (Fig. 7C, D, E). In addition, compared to ox-LDL group, the expression of phosphorylation of DRP1 (ser616) at protein level was decreased by 50 μM Mdivi-1 but DRP1 expression in total cell did not significantly change (Fig. 7F, G, H). In addition, the translocation of DRP1 from cytoplasm to mitochondria (Fig. 7I, J, K) was then significantly reduced. These results indicated that Mdivi-1 treatment could reduce excessive mitochondrial fission and improve mitochondrial dysfunction by inhibiting DRP1 translocation from cytoplasm to mitochondria via supressing the phosphorylation of DRP1 at Ser616, which reduced the production of mito-ROS and then inhibited M1 polarization by suppressing the NLRP3 activiation.

### The gene *DRP1* knockdown reduces M1 polarization-mediated foam cell formation by suppressing the mito-ROS/NLRP3 pathway

**Fig. 8:**
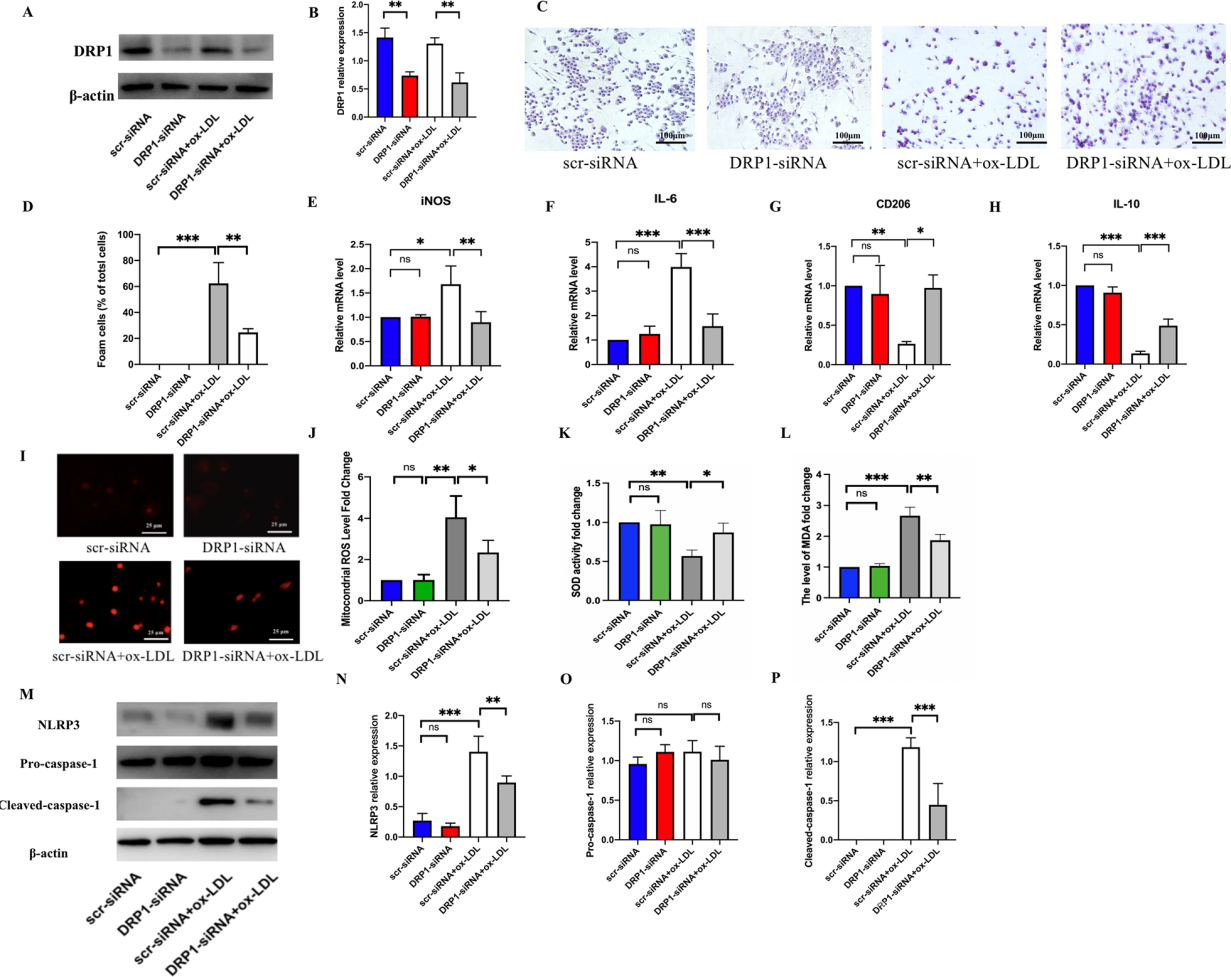
DRP1 knockdown reduced M1 polarization mediated foam cell formation by inhibiting mito-ROS/NLRP3 activation. **A** The protein level of DRP1 detected by western blot. The relative protein expression levels of (**B**) DRP1/β-actin in RAW264.7 cells. **C**, **D** ORO staining to detect lipid deposition in macrophages (scale bar: 100 μm. 200 × magnification). qRT-PCR to detect the mRNA expression of M1 polarization markers, including (**E**) iNOS, (**F**) IL-6 and M2 markers, including (**G**) CD206, and (**H**) IL-10. **I**, **J** mito-ROS detected by mito-SOX (scale bar: 25 μm. 200 × magnification). (**K**, **L**) The SOD activity and level of MDA detected by corresponding kits. **M** The protein level of NLRP3, pro-caspase-1, and cleaved-caspase-1 detected by western blot. The relative protein expression levels of (**N**) NLRP3/β-actin (**O**) pro-caspase-1/β-actin and (**P**) cleaved-caspase-1/β-actin in RAW264.7 cells. Three independent replications were performed. * *p* < 0.05, ** *p* < 0.01, *** *p* < 0.001, and ns *p* > 0.05

Here, we investigated the role of *DRP1* knockdown on M1 polarization and its probable underlying mechanism. The expression of *DRP1* at protein level was decreased by DRP1-siRNA treatment (Fig. 8A, B). ORO Red staining (Fig. 8C) and percent positive foam cells (Fig. 8D) revealed that *DRP1* knockdown significantly attenuated foam cell formation by reducing lipid droplets deposition. Then, the results showed that the *DRP1* knockdown downregulated the mRNA level of M1 markers (iNOS and IL-6), but upregulated the mRNA level of M2 markers (CD206 and IL-10) in the ox-LDL-stimulated macrophages (Fig. 8E–H). In addition, *DRP1* knockdown also reduced mito-ROS, MDA accumulation with an increased activity of SOD (Fig. 8I–L) and inhibited NLRP3 activation (Fig. 8M–P). These results suggested that downregulating *DRP1* expression also alleviated M1 polarization mediated foam cell formation by suppressing the mito-ROS/NLRP3 pathway.

### *DRP1* knockdown improved mitochondrial fission associated mitochondrial dysfunction induced by ox-LDL

**Fig. 9:**
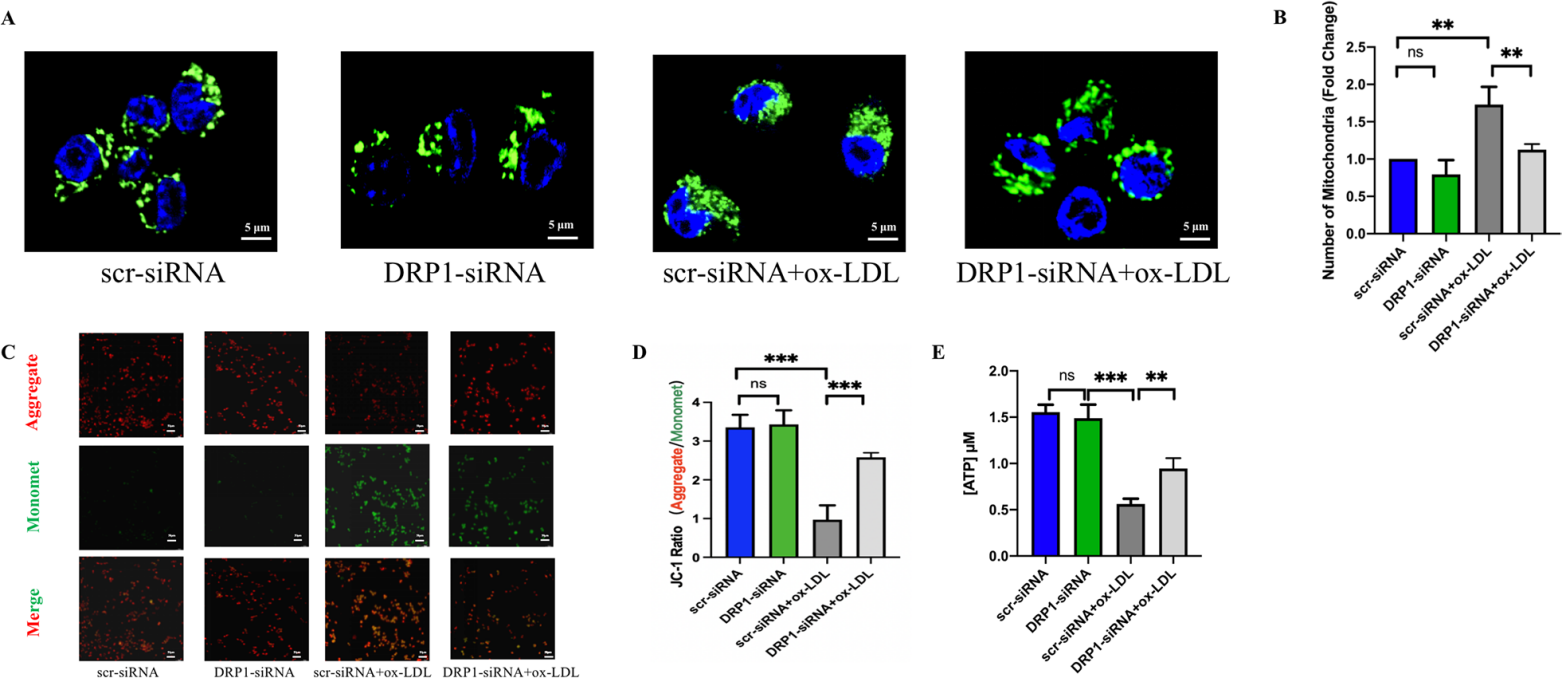
DRP1 knockdown improved mitochondrial fission associated mitochondrial dysfunction caused by ox-LDL. **A** MitoTracker staining to detect the number of mitochondrial fragments (scale bar: 5 μm. 600 × magnification). **B** Analysis of the relative number of mitochondria. **C**, **D** JC-1 staining to detect mitochondrial membrane potential (scale bar: 50 μm. 200 × magnification). **E** Detecting ATP level by an ATP determination kit. Three independent replications were performed. ** *p* < 0.01, *** p < 0.001, and ns *p* > 0.05

Our results showed that the division of mitochondria was increased in scr-siRNA + ox-LDL group, and *DRP1* knockdown could reduce the number of mitochondria by inhibiting fission (Fig. 9A, B). Unmerged figures are shown in Additional file 4: Fig. S3. In addition, JC-1 ratio and the ATP level were decreased after ox-LDL treatment, whereas, *DRP1* knockdown improved the function of mitochondria by ameliorating mitochondrial membrane potential and ATP synthesis (Fig. 9C, D, E). These results indicated that *DRP1* knockdown could improve excessive mitochondrial fission associated mitochondrial dysfunction, then inhibiting M1 polarization via mito-ROS/NLRP3 pathway.

## Discussion

**Fig. 10:**
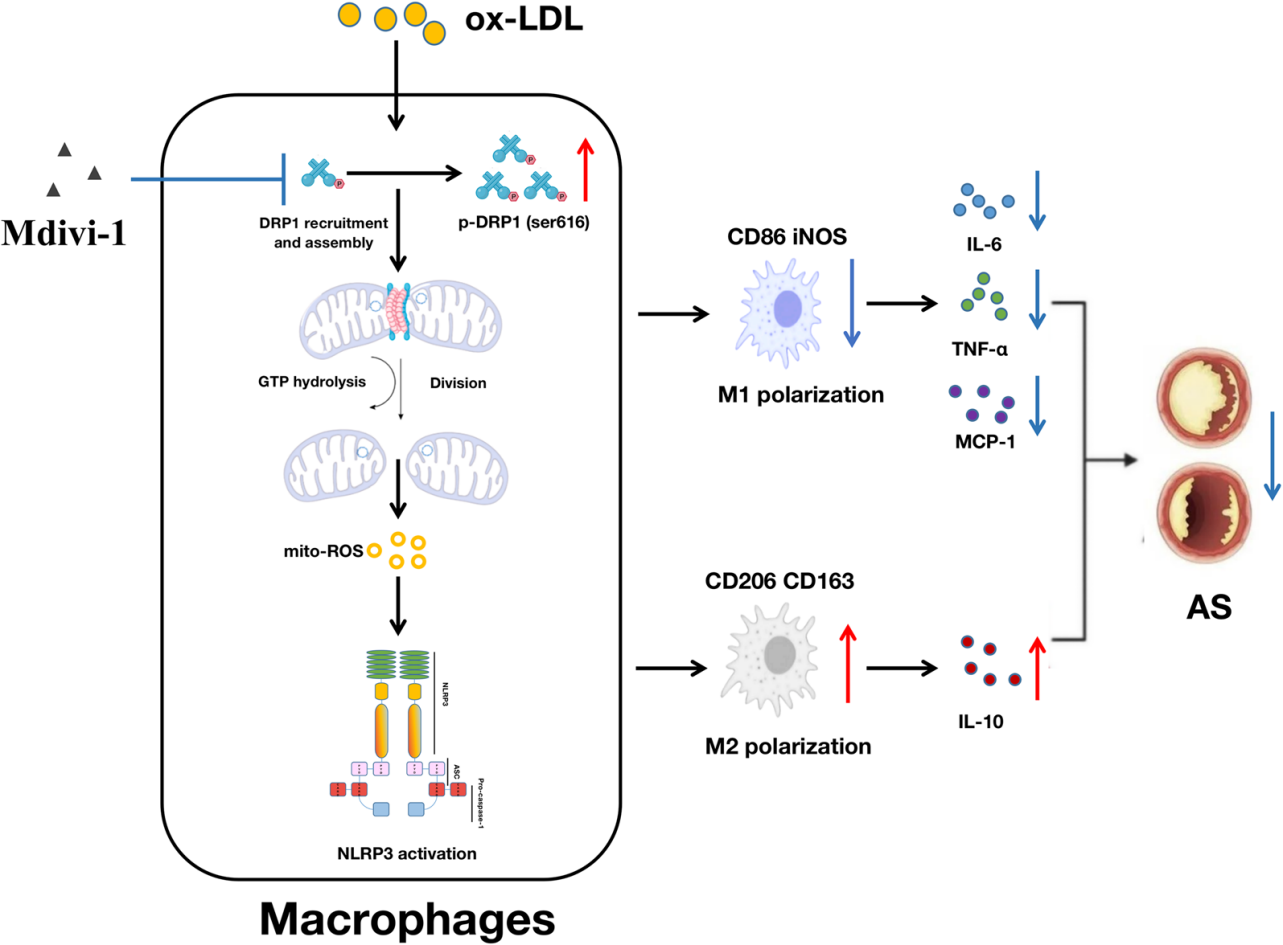
Mechanism diagram of the anti-atherosclerotic effect of DRP1-dependent mitochondrial fission inhibition by Mdivi-1

In this study, we explored the possible role of DRP1-dependent mitochondrial fission in affecting the processes of M1 polarization and subsequently mitigating AS, both in vivo and in vitro. Our data demonstrated that the DRP1 inhibitor, Mdivi-1, could effectively alleviate AS plaques by inhibiting M1 polarization. Furthermore, the possible mechanism responsible for the anti-AS effects of mitochondrial fission inhibition by Mdivi-1 on reducing M1 polarization could be associated with the suppression of the mito-ROS/NLRP3 pathway (Fig. 10).

Atherogenesis is caused by abnormal lipid accumulation in the arterial wall, which is regarded as a chronic inflammation disorder [40]. During hyperlipidemia, endothelial injury plays a significant role in the abnormal accumulation of ox-LDL and promotes monocyte migration from the bloodstream into the arterial wall. Lipid metabolism disorder in the intima differentiates monocytes into macrophages, then engulfs ox-LDL into foam cells, ultimately triggering atherosclerosis [41]. Currently, the modulation of macrophage polarization is a novel target for AS. ox-LDL could induce macrophages to M1 polarization with high expression of MI markers (CD86, iNOS, IL-6, TNF-α, and MCP-1) and decreased M2 markers (CD206, CD163 and IL-10), which promoted plaque progression [3, 4]. Targeted inhibition of M1 polarization and reducing pro-inflammatory cytokines released by M1 could significantly alleviate AS [42, 43]. This present study showed that Mdivi-1 intervention inhibited HFD-driven inflammation and atheroma plaques in ApoE^−/−^ mice by suppressing M1 polarization. In vitro, Mdivi-1 treatment and downregulating the DRP1 could suppress foam cell formation by inhibiting M1 polarization in RAW264.7 cells stimulated by ox-LDL.

Regulating NLRP3 might be a potential target to protect against atherogenesis [15, 28, 44, 45]. Recently, NLRP3 inflammasome also played a crucial role in various inflammation-based disorders by regulating M1 polarization [17, 31, 32, 46, 47]. However, whether inhibiting NLRP3 inflammasome activation could suppress AS by manipulating M1 polarization is poorly understood. In this present study, M1 polarization and NLRP3 activation were found in the aortic tissues of HFD mice. Similar results were also found in vitro, suggesting that M1 macrophage polarization and NLPR3 activation participated in AS. Further, the specific small-molecule NLRP3 inhibitor, MCC950, could significantly inhibit ox-LDL inducing M1 polarization and associated foam cell formation. Taken together, our results showed that NLRP3-dependent M1 polarization might play a significant role in AS. Previous literature reported that the aggregation and activation of NLRP3 inflammasome were regulated by extracellular ATP, extracellular crystals, and abnormal accumulation of intracellular ROS, which were mainly released by dysfunctional mitochondria [48]. As an indispensable risk factor for AS, ox-LDL could significantly induce inflammation and foam cell formation with the elevated level of ROS in macrophages [49, 50]. Improving the abnormal accumulation of ROS was shown to inhibit ox-LDL uptake by macrophages [51]. In this study, we found that the mito-ROS scavenger Mito-TEMPO could reduce M1 polarization-mediated foam cell formation by inhibiting NLRP3 activation, indicating that mito-ROS/NLRP3 dependent M1 polarization could promote AS.

It has been shown that mitochondrial dysfunction can lead to the initiation and progression of AS [52, 53]. Also, increasing evidence emphasized that mitochondrial homeostasis is related to mitochondrial fusion/fission [54, 55]. DRP1 is also an indispensable core molecule controlling mitochondrial fission [37]. DRP1 activity can be reversibly modified by two critical phosphorylation sites. Phosphorylation of DRP1 at serine 616 promotes DRP1 activity, which can stimulate DRP1 mitochondrial translocation and promote fission. Conversely, phosphorylation of serine 637 represses its activity and hinder DRP1 recruitment to the mitochondria then inhibiting fission [37]. Recently, Mdivi-1 could protect against cardiac fibrosis post myocardial infarction [56] and angiotensin-II-induced hypertension [25] by suppressing phosphorylation of DRP1 at serine 616 and associated mitochondrial fission. Furthermore, the inhibition of DRP1-dependent mitochondrial fission in endothelial cells exerted an anti-atherosclerotic effect [14, 22]. However, the potential relationship between mitochondrial fission and macrophage polarization has not been reported in AS. Our study showed that Mdivi-1 effectively suppressed the expression of phospho-DRP1(Ser616) and alleviated atheroma plaques by inhibiting M1 polarization in vivo. In addition, the inhibition of phospho-DRP1(Ser616) by Mdivi-1 reduced the translocation of DRP1 into mitochondria then reducing the division of mitochondria and associated mitochondrial dysfunction induced by ox-LDL, which then reduced M1 polarization mediated foam cell formation in vitro. Moreover, the similar results were observed by *DRP1* knockdown. Therefore, Mdivi-1 could effectively inhibit the activity of DRP1 by restraining phospho-DRP1(Ser616) then reducing excessive mitochondrial fission, which alleviated M1 polarization mediated AS.

Macrophages are classical innate immune cells acting as danger-sensing sentinels to ensure immunological homeostasis [57, 58]. Inflammation and immune dysfunction with macrophage infiltration are critically important mechanisms in AS [59]. Mitochondria have many functions, including energy generation, cell signaling, ROS synthesizing, and more [60]. It was shown that mitochondria regulate innate immune responses, ROS production and the homeostasis and inflammatory status of macrophages [61], and mitochondrial fission regulated the inflammation of macrophages. Kapetanovic et al. showed lipopolysaccharide-induced inflammatory responses in macrophages via DRP1-dependent mitochondrial fission [62]; Yu et al. reported that the pro-inflammatory differentiation of macrophages was associated with DRP1-mediated mitochondrial mass increase [26]. In regard to AS, DRP1-mediated mitochondrial fission was related to oxidative stress and pro-inflammatory responses of ox-LDL-induced macrophages [63]. In this study, we found that inhibiting phospho-DRP1(Ser616) by Mdivi-1 provided protection against AS plaques by inhibiting NLRP3 activation and M1 polarization in the aortic tissues of HFD mice. In addition, inhibition of DRP1-dependent fission by Mdivi-1 or *DRP1* knockdown significantly reduced the abnormal accumulation of mito-ROS and inhibited NLRP3 activation, then decreasing M1 polarization mediated foam cell formation in ox-LDL induced macrophages. Taken together, our results showed that Mdvi-1 could inhibit DRP1-dependent mitochondrial fission then reducing M1 macrophage polarization mediated atherogenesis by regulating the mito-ROS/NLRP3 inflammasome singling pathway.

## Limitation

Only one cell line, RAW264.7, was used in this study, and the results might not perfectly reflect the exact role of mitochondrial fission inhibition by Mdivi-1 in alleviating AS. Thus, future studies should include other macrophage cell lines, such as THP-1, to further investigate the regulation of mitochondrial fission in M1 polarization-associated AS. In addition, the role of DRP1 inhibiting in the division of mitochondria and modulating M1 polarization-mediated AS should be further explored by knocking down DRP1 in vivo.The study was conducted on mice and in vitro cell cultures, so the results may not be directly applicable to humans. Only one DRP1 inhibitor (Mdivi-1) was used in the study; it is possible that other inhibitors or methods for inhibiting DRP1-dependent mitochondrial fission would have different effects.

## Conclusion

DRP1-dependent mitochondrial fission induces M1 polarization to accelerate atherogenesis by regulating the mito-ROS/NLRP3 inflammasome singling pathway. Thus, targeting DRP1-dependent mitochondrial fission by Mdivi-1 may protect against atherosclerosis.

## Supplementary Information

**Additional file 1 : Table S1.** Primer sequences used for qRT-PCR analysis.

**Additional file 2 :** **Figure S1.** Effects of Mdivi-1 on mitochondrial fission inhibition in aortic structure in HFD treated ApoE^-/-^ mice (scale bar: 2μm. 8000×magnification).

**Additional file 3 :** **Figure S2.** Unmerged **Figure 7A:** MitoTracker staining to detect the number of mitochondrial fragments (scale bar: 5μm. 600×magnification).

**Additional file 4 :** **Figure S3.** Unmerged **Figure 9A:** MitoTracker staining to detect the number of mitochondrial fragments (scale bar: 5μm. 600×magnification).

## Data Availability

The original contributions presented in the study are included in the article. Further inquiries can be directed to the corresponding author.
