# Supplementary material for: Inhibition of DRP1-dependent mitochondrial fission by Mdivi-1 alleviates atherosclerosis through the modulation of M1 polarization
Source: J Transl Med. 2023 Jun 30;21:427. doi: 10.1186/s12967-023-04270-9 (PMC10311781; doi:10.1186/s12967-023-04270-9)
Supplement: Supplementary file 1 — Additional file 1 : Table S1. Primer sequences used for qRT-PCR analysis. [file 12967_2023_4270_MOESM1_ESM.docx]

**Table 1. Primer sequences used for qRT-PCR analysis.**

| **Promotor of gene** | **Forward primer** | **Reverse primer** |
| --- | --- | --- |
| β-actin | 5'-AGCATGGTGCCACTGTGCT-3' | 5'-GGCTTAATCTCTGCCTCATGAT-3' |
| TNF-α | 5'-TCAACCTCCTCTCTGCCGT-3' | 5'-CTCCAAAGTAGACCTGCCCG-3' |
| IL-6 | 5'-TCTTGGGACTGATGCTGGTG-3’ | 5'-AGGTCTGTTGGGAGTGGTATC-3’ |
| iNOS | 5'-ACGAGACGGATAGGCAGAGA-3' | 5'-GCTGATGGAGTAGTAGCGGG-3' |
| CD86 | 5'-ATCCAAGAGCCACTCCTACCT-3’ | 5'-TCCAGACCTTTCCAGGCATTT-3’ |
| MCP-1 | 5'-CCACAACCACCTCAAGCACT-3' | 5'-AAGGCATCACAGTCCGAGTCA-3' |
| CD163 | 5'-TGCTCAGGAAACCAATCCCAG-3' | 5'-ACCTCCACCTACCAAGCGAA-3' |
| CD206 | 5'-GATAGATGGAGGGTGCGGTA-3' | 5'-GAGGAGGTTCAGTAGCAGGG-3' |
| IL-10 | 5'-GCACTACCAAAGCCACAAGG-3' | 5'-TGCCAGTCAGTAAGAGCAGG-3' |
