# Supplementary figures and images for: Inhibition of DRP1-dependent mitochondrial fission by Mdivi-1 alleviates atherosclerosis through the modulation of M1 polarization
Source: J Transl Med. 2023 Jun 30;21:427. doi: 10.1186/s12967-023-04270-9 (PMC10311781; doi:10.1186/s12967-023-04270-9)

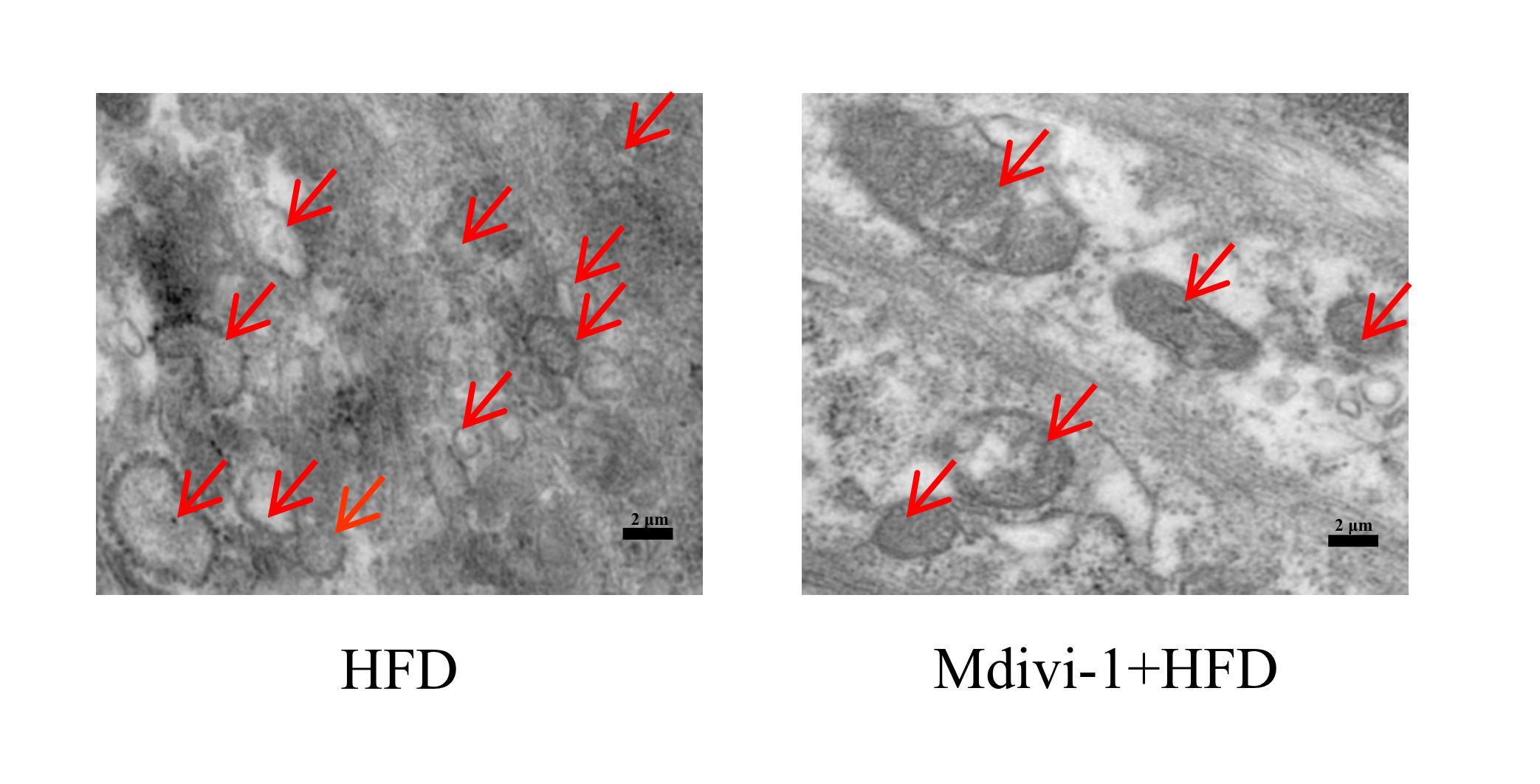

Supplement: Supplementary file 2 — Additional file 2 : Figure S1. Effects of Mdivi-1 on mitochondrial fission inhibition in aortic structure in HFD treated ApoE-/- mice (scale bar: 2μm. 8000×magnification). [file 12967_2023_4270_MOESM2_ESM.tif]

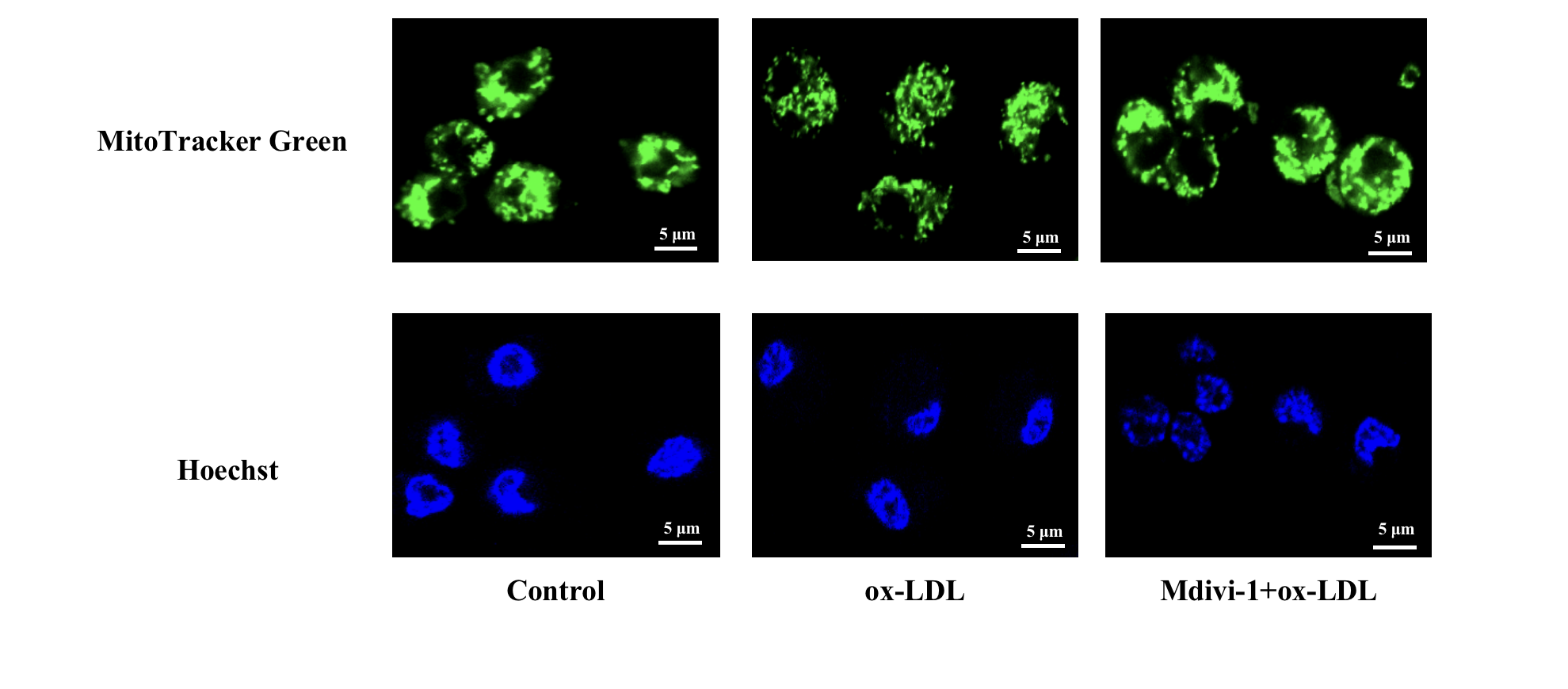

Supplement: Supplementary file 3 — Additional file 3 : Figure S2. Unmerged Figure 7A: MitoTracker staining to detect the number of mitochondrial fragments (scale bar: 5μm. 600×magnification). [file 12967_2023_4270_MOESM3_ESM.tif]

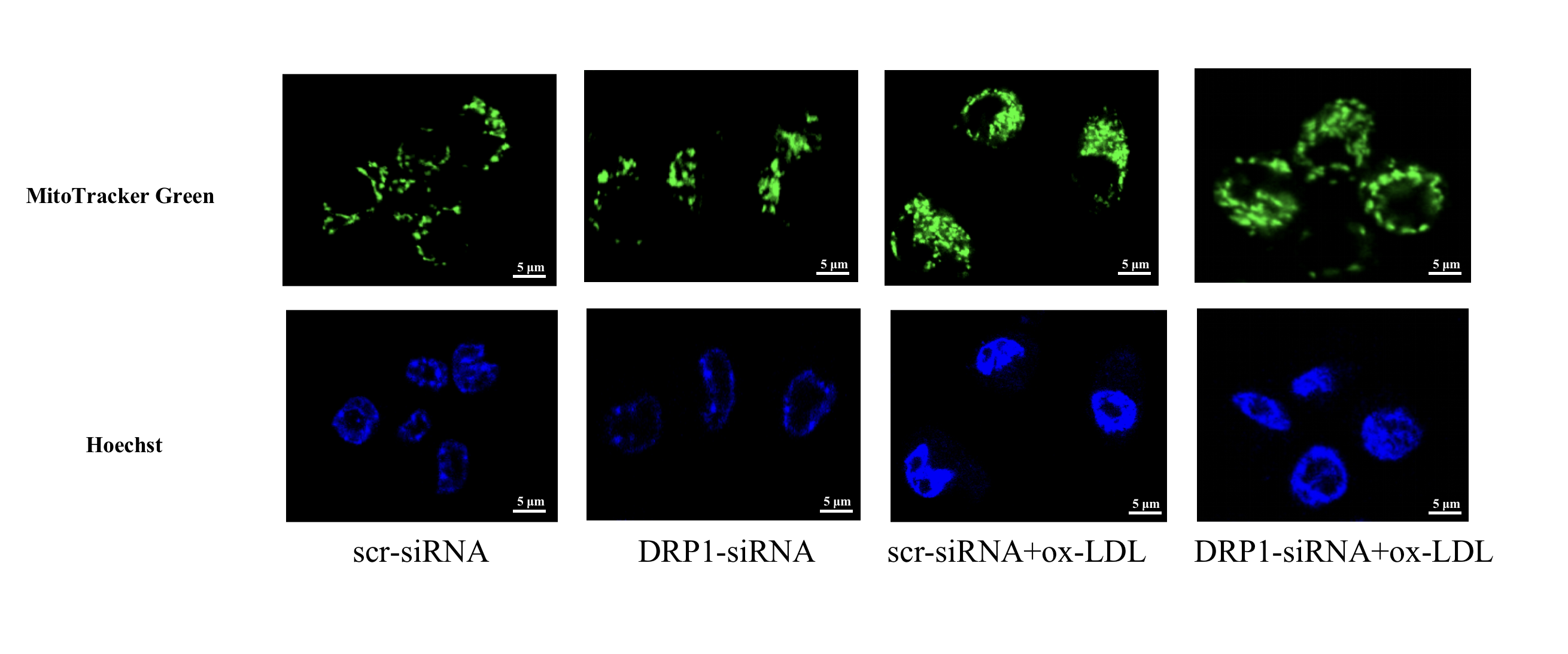

Supplement: Supplementary file 4 — Additional file 4 : Figure S3. Unmerged Figure 9A: MitoTracker staining to detect the number of mitochondrial fragments (scale bar: 5μm. 600×magnification). [file 12967_2023_4270_MOESM4_ESM.tif]
